# Women in Medicine, Law and Theology

**Published:** 1875-11

**Authors:** 


Women in Medicine, Law and Theology.—Amongst the burning questions of the day is the propriety or the right of admitting women into the professions. I say the right or propriety, because even if the right were made good, the propriety would not follow. Why it is that the women have selected medicine as the especial point of attack is not quite clear. They are far better fitted by organization, by natural aptitude, to shine in the pulpit and at the bar. And in the career of arms, if we may trust legendary or mystic history, they have earned no mean distinction. But medicine, whilst demanding physical power not less than the other professions, is essentially based upon science.

Now, the women who have been distinguished for scientific power might be counted on the fingers of one hand. There seems to be a natural incompatibility between science and the female brain. There is, indeed, no such inherent incompatibility between science and religion or law. Theology and law that are not in harmony with true science must rest upon a very insecure foundation. But the clergy of all denominations, and lawyers as a rule, assume a direct antagonism to science. They set themselves above it; they would trample it down as something in chronic rebellion against their authority. In this antagonism they represent the women; in these they find their most useful allies. The church and the law, then, are the professions most congenial to the somewhat arbitrary character of the female intellect.—*Obstetrical Journal, August,* 1875.

